# Integrated In Vitro and In Silico Evaluation of Benzimidazolium Salts: Antioxidant Activity, Anti-MRSA/MSSA Effects, and Antibacterial Gene Expression Analysis

**DOI:** 10.3390/antibiotics15060567

**Published:** 2026-06-02

**Authors:** Başak Bedir, Hakan Ünver, Mehmet Çimentepe, Özge Öztürk Çimentepe, Akın Yiğin, Metin Yildirim

**Affiliations:** 1 Department of Nutrition and Dietetics, Faculty of Health Sciences, Hakkari University, Hakkâri 30000, Türkiye; 2 Department of Chemistry, Faculty of Science, Eskisehir Technical University, Eskisehir 26555, Türkiye; hakanunver@eskisehir.edu.tr; 3 Department of Pharmaceutical Microbiology, Faculty of Pharmacy, Harran University, Sanliurfa 63300, Türkiye; mehmet.cimentepe@harran.edu.tr; 4 Department of Pharmacology, Faculty of Pharmacy, Harran University, Sanliurfa 63300, Türkiye; ozge.cimentepe@harran.edu.tr; 5 Department of Genetics, Faculty of Veterinary Medicine, Harran University, Sanliurfa 63200, Türkiye; akinyigin@harran.edu.tr; 6 Department of Biochemistry, Faculty of Pharmacy, Cukurova University, Adana 01330, Türkiye

**Keywords:** benzimidazolium derivatives, MRSA, molecular docking, gene expression, antibacterial activity

## Abstract

Background/Objectives: The emergence of methicillin-resistant *Staphylococcus aureus* (MRSA) has created an urgent need for the development of novel antimicrobial agents. This study aimed to synthesize and characterize a series of benzimidazolium salts and evaluate their antibacterial, antibiofilm, and molecular interaction properties against MRSA and methicillin-susceptible *Staphylococcus aureus* (MSSA). Methods: Five benzimidazolium salts bearing different substituents were synthesized and characterized by NMR and FTIR analyses. Their antibacterial activities against MRSA and MSSA were investigated using the resazurin-based minimum inhibitory concentration (MIC) assay and agar diffusion method. Antibiofilm activity was evaluated using crystal violet staining, while scanning electron microscopy (SEM) was employed to examine bacterial morphological changes. Gene expression analysis was performed to assess the effects of the most active compound on virulence- and resistance-related genes. In addition, molecular docking studies were conducted against four target proteins (1MWT, 3ZG5, 1JIJ, and 2Y2M). Results: Among the synthesized compounds, compound 1c exhibited the strongest antibacterial activity, with MIC values of 0.25 µg/mL against MRSA and 0.5 µg/mL against MSSA. It also produced the largest inhibition zone diameter (31.0 ± 0.5 mm). SEM analysis revealed significant morphological alterations in treated bacterial cells, indicating disruption of cellular integrity. Gene expression studies demonstrated that compound 1c downregulated several virulence- and resistance-associated genes, including *icaA*, *dltA*, *dltB*, *sarA*, *norA*, and *norB*. All compounds displayed antibiofilm activity, with compound 1c showing the highest inhibition rate (68.6 ± 0.6%). Molecular docking analysis revealed that compound 1c exhibited the strongest binding affinity toward the 2Y2M target protein, with a docking score of −5.322 kcal/mol. Conclusions: The findings demonstrate that benzimidazolium derivatives, particularly compound 1c, possess potent antibacterial and antibiofilm activities against *S. aureus* strains and effectively modulate virulence- and resistance-related gene expression. Combined with favorable molecular docking results, these compounds represent promising candidates for the development of new therapeutic agents against resistant S. aureus infections.

## 1. Introduction

The global rise of antimicrobial resistance (AMR) has become one of the most critical challenges in modern healthcare due to its ability to reduce the effectiveness of conventional antimicrobial therapies [1]. The overuse and misuse of antibiotics have paved the way for the emergence and dissemination of resistant bacterial strains which lead to increased morbidity, mortality, and a substantial global healthcare burden [2]. *Staphylococcus aureus* is a major clinical threat among these pathogens because it causes a broad spectrum of infections, from superficial skin lesions to life-threatening systemic diseases [3]. This pathogen has become a leading cause of both hospital- and community-acquired infections since the emergence of methicillin-resistant *S. aureus* (MRSA) in the 1960s. In addition, it is characterized by limited therapeutic options and frequent treatment failure even with last-line agents such as vancomycin and linezolid [4,5]. However, these limitations underscore the urgent need for novel, mechanism-based antimicrobial strategies.

This clinical burden is driven by complex molecular mechanisms enabling MRSA survival under antimicrobial pressure. The methicillin-resistant phenotype is primarily mediated by the *mecA* gene encoding PBP2a, which exhibits reduced affinity for β-lactam antibiotics [6,7]. However, MRSA pathogenicity is not governed by a single determinant but by an integrated resistance–virulence network [8]. This system includes β-lactam resistance (*mecA*), efflux-mediated adaptation (*mepA*), and regulatory pathways controlling virulence and persistence (*sarA*, *icaA*, *dltB*). These elements, together, coordinate bacterial survival under antimicrobial exposure and host immune stress [9,10]. The specific contributions of these regulators are outlined below. Within this coordinated network, *sarA* regulates virulence and biofilm development, while the ica operon drives production of polysaccharide intercellular adhesin (PIA), a key component of the biofilm matrix [11,12]. In addition, *dltB* contributes to cell envelope modification, enhancing resistance to host defense peptides, whereas *mepA* mediates multidrug efflux and adaptive stress responses, which contributes to antimicrobial resistance dynamics [13,14].

Collectively, these mechanisms promote MRSA persistence by augmenting biofilm formation, a key determinant of chronic infection. Biofilms consist of bacteria embedded in a self-produced matrix that reduces antibiotic penetration and increases metabolic dormancy, resulting in high tolerance and recurrent infections [15]. Therefore, simultaneous targeting of resistance pathways and biofilm formation is essential for next-generation antimicrobial strategies [16].

Within this context, benzimidazolium salts represent a multifunctional bioactive structure with significant antimicrobial properties. In this research, they acted as the only active framework to enable a targeted understanding of structure–activity relationships. These cationic compounds interact electrostatically with bacterial membranes and internal targets, and their effectiveness can be adjusted through structural changes like N-alkylation and counterion variation. However, the influence of these factors on MRSA regulatory networks remains only partially understood, necessitating a combination of phenotypic and genotypic approaches. To attain a comprehensive understanding of MRSA, it is imperative to correlate antimicrobial effects with molecular responses. Together, these molecular targets constitute a biologically meaningful framework for evaluating the capacity of novel antimicrobial candidates to simultaneously disrupt multiple survival pathways, rather than merely exerting conventional bacteriostatic or bactericidal effects [17,18,19,20]. Guided by this rationale, compounds capable of modulating these factors may enhance understanding of both antibacterial efficacy and the underlying mechanisms of action.

Accordingly, in this study, benzimidazolium salts were employed as the bioactive scaffold. Novel benzimidazolium salt derivatives were synthesized and structurally characterized, followed by evaluation of antibacterial and antibiofilm activities against MSSA and MRSA strains. Gene expression profiling and molecular docking analyses were then performed to elucidate mechanistic interactions underlying the observed biological effects.

## 2. Materials and Methods

### 2.1. Materials and Equipment

No further purification with solvents or reagents was required. 1-(4-Methylbenzyl)-2-phenyl-1*H*-benzo[*d*]imidazole (Compound **1**) was isolated by precipitation in water, subsequently dried, and used directly without additional purification, in agreement with previously reported data [21]. (Bromomethyl)benzene (TCI, Tokyo, Japan), 1-bromo-4-(bromomethyl)benzene (TCI, Tokyo, Japan), 1-(bromomethyl)-4-(trifluoromethyl)benzene (TCI, Tokyo, Japan), 4-(bromomethyl)benzoic acid (TCI, Tokyo, Japan), and methyl 4-(bromomethyl)benzoate (Apollo Scientific, Stockport, England), were obtained commercially. ^1^H and ^13^C NMR spectra were recorded on an Agilent 400 MHz FT-NMR spectrometer (Agilent Technologies, Santa Clara, CA, USA).

### 2.2. Methods

#### 2.2.1. Synthesis Procedure of Benzimidazolium Salts

A solution of Compound **1** (200 mg, 0.56 mmol, 1 equiv.) was prepared in 20 mL of acetonitrile (CH_3_CN) under ambient conditions. The appropriate benzyl bromide (0.56 mmol, 1 equiv.) was then added portionwise. The resulting mixture was maintained at 70 °C and stirred continuously for 48 h. After complete precipitation was observed and the completion of the reaction was confirmed by TLC (EtOAc/hexane = 1:4), the resulting solid was collected by filtration, washed with a small amount of diethyl ether to remove residual impurities, and subsequently dried under reduced pressure to afford the desired product as a solid.

**3-benzyl-1-(4-methylbenzyl)-2-phenyl-1*H*-benzo[*d*]imidazol-3-ium bromide (Compound 1):**

White solid, Yield: 78%. ^1^H-NMR (400 MHz, DMSO-*d*_6_) *δ* (ppm): 7.99 (dq, *J* = 6.0, 3.1, 2.6 Hz, 2H), 7.87 (d, *J* = 7.7 Hz, 2H), 7.81–7.74 (m, 1H), 7.69 (ddd, *J* = 13.0, 7.6, 3.1 Hz, 4H), 7.29 (d, *J* = 4.9 Hz, 3H), 7.19–7.14 (m, 2H), 7.10 (d, *J* = 7.8 Hz, 2H), 7.08–7.02 (m, 2H), 5.60 (s, 2H), 5.54 (s, 2H), 2.23 (s, 3H). ^13^C-NMR (100 MHz, DMSO-*d*_6_) *δ* (ppm): 151.44, 138.21, 134.31, 133.61, 131.72, 131.67, 131.27, 130.84, 130.13, 129.80, 129.26, 128.80, 127.65, 127.48, 121.44, 114.60, 114.53, 49.67, 49.53, 21.10. FT-IR (KBr, cm^−1^): 3036, 2921, 1615.

**3-(4-bromobenzyl)-1-(4-methylbenzyl)-2-phenyl-1*H*-benzo[*d*]imidazol-3-ium bromide (Compound 2):**

White solid, Yield: 75%. ^1^H-NMR (400 MHz, DMSO-*d*_6_) *δ* (ppm): 7.98 (dd, *J* = 6.7, 3.5 Hz, 2H), 7.83 (d, *J* = 7.7 Hz, 2H), 7.77 (d, *J* = 7.7 Hz, 1H), 7.72–7.65 (m, 4H), 7.50 (dd, *J* = 8.5, 2.3 Hz, 2H), 7.15–7.08 (m, 4H), 7.08–7.02 (m, 2H), 5.57 (s, 2H), 5.53 (s, 2H), 2.23 (s, 3H). ^13^C-NMR (100 MHz, DMSO-*d*_6_) *δ* (ppm): 151.56, 138.21, 133.78, 133.65, 132.11, 131.70, 131.23, 130.78, 130.14, 129.89, 129.79, 129.03, 127.66, 127.55, 127.51, 126.93, 122.03, 121.35, 114.61, 114.44, 110.00, 49.53, 49.06, 21.09. FT-IR (KBr, cm^−1^): 3052, 2933, 1615.

**1-(4-methylbenzyl)-2-phenyl-3-(4-(trifluoromethyl)benzyl)-1*H*-benzo[*d*]imidazol-3-ium bromide (Compound 3):**

White solid, Yield: 69%. ^1^H-NMR (400 MHz, DMSO-*d*_6_) *δ* (ppm): 7.99 (d, *J* = 6.8 Hz, 2H), 7.84 (d, *J* = 7.7 Hz, 2H), 7.77 (t, *J* = 7.6 Hz, 1H), 7.68 (t, *J* = 5.8 Hz, 6H), 7.40 (d, *J* = 7.9 Hz, 2H), 7.11 (d, *J* = 8.0 Hz, 2H), 7.07 (d, *J* = 7.8 Hz, 2H), 5.71 (s, 2H), 5.54 (s, 2H), 2.23 (s, 3H). ^13^C-NMR (100 MHz, DMSO-*d*_6_) *δ* (ppm): 151.70, 139.08, 138.22, 133.66, 131.77, 131.73, 131.22, 130.76, 130.13, 129.79, 129.36, 129.04, 128.42, 127.68, 127.62, 127.57, 126.08, 126.05, 125.79, 121.30, 114.65, 114.38, 110.00, 49.59, 49.16, 21.10. FT-IR (KBr, cm^−1^): 3033, 2915, 1619.

**3-(4-carboxybenzyl)-1-(4-methylbenzyl)-2-phenyl-1*H*-benzo[*d*]imidazol-3-ium bromide (Compound 4):**

White solid, Yield: 71%. ^1^H-NMR (400 MHz, DMSO-*d*_6_) *δ* (ppm): 13.06 (s, 1H), 7.98 (d, *J* = 6.8 Hz, 2H), 7.83 (t, *J* = 6.7 Hz, 4H), 7.75 (d, *J* = 7.6 Hz, 1H), 7.68 (d, *J* = 7.4 Hz, 4H), 7.27 (d, *J* = 8.1 Hz, 2H), 7.11 (d, *J* = 7.9 Hz, 2H), 7.06 (d, *J* = 7.8 Hz, 2H), 5.68 (s, 2H), 5.54 (s, 2H), 2.23 (s, 3H). ^13^C-NMR (100 MHz, DMSO-*d*_6_) *δ* (ppm): 167.23, 151.65, 139.18, 138.21, 133.64, 131.81, 131.70, 131.24, 131.08, 130.75, 130.12, 130.09, 129.97, 129.80, 127.72, 127.65, 127.59, 127.55, 121.32, 114.64, 114.40, 49.57, 49.39, 21.10. FT-IR (KBr, cm^−1^): 3032, 2918, 1614.

**3-(4-(methoxycarbonyl)benzyl)-1-(4-methylbenzyl)-2-phenyl-1H-benzo[d]imidazol-3-ium bromide (Compound 5):**

White solid, Yield: 73%. ^1^H-NMR (400 MHz, DMSO-*d*_6_) *δ* (ppm): 7.99 (dd, *J* = 6.3, 3.1 Hz, 2H), 7.84 (dd, *J* = 15.9, 7.8 Hz, 4H), 7.76 (t, *J* = 7.5 Hz, 1H), 7.67 (dt, *J* = 9.4, 5.6 Hz, 4H), 7.30 (d, *J* = 7.9 Hz, 2H), 7.11 (d, *J* = 7.9 Hz, 2H), 7.06 (d, *J* = 7.9 Hz, 2H), 5.70 (s, 2H), 5.54 (s, 2H), 3.81 (s, 3H), 2.23 (s, 3H). ^13^C-NMR (100 MHz, DMSO-*d*_6_) *δ* (ppm): 166.17, 151.67, 139.68, 138.21, 133.64, 131.84, 131.70, 131.23, 130.74, 130.11, 129.94, 129.88, 129.80, 129.26, 127.90, 127.66, 127.60, 127.56, 121.30, 114.65, 114.38, 52.73, 49.58, 49.36, 21.10. FT-IR (KBr, cm^−1^): 3022, 2921, 1614.

**Figure 1:**
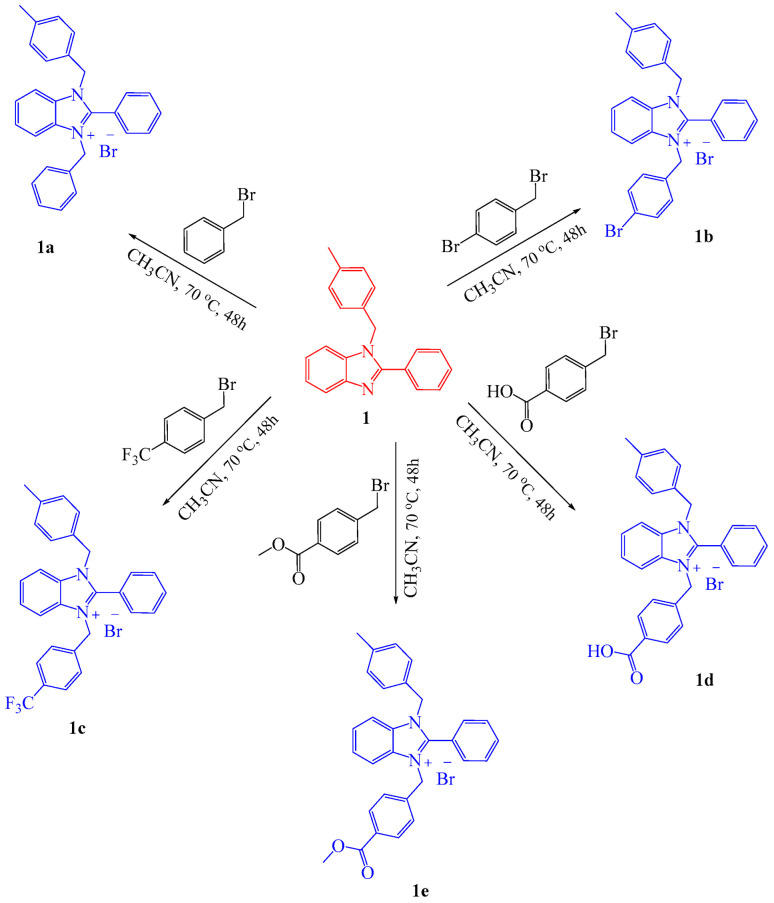
Synthesis procedure of 1-(4-methylbenzyl)-2-phenyl-1*H*-benzo[*d*]imidazolium salts.

The syntheses scheme of 1-(4-methylbenzyl)-2-phenyl-1*H*-benzo[*d*]imidazolium salts is given in Figure 1.

#### 2.2.2. Antimicrobial Activity

The antimicrobial activities of compounds **1a**–**1e** against MSSA (ATCC 25923) and MRSA (ATCC 43300) strains were determined using the resazurin-based microdilution assay and the agar well diffusion method [22,23].

In the broth microdilution method, 100 μL of sterile Mueller–Hinton Broth was first dispensed into each well. Subsequently, 100 μL of the compounds at a concentration of 256 μg/mL was added to the first well, followed by a two-fold serial dilution across the plate. Then, 100 μL of bacterial suspension with an approximate density of 1 × 10^6^ CFU/mL was added to the respective wells. As a result, the final bacterial concentration reached 5 × 10^5^ CFU/mL, while the compound concentrations ranged between 0.25 and 128 μg/mL. For controls, 100 μL of sterile medium was used as the negative control, and 100 μL of bacterial suspension served as the growth control. After incubation at 37 °C for 16–18 h, 20 μL of 0.01% resazurin solution was added to each well and incubated briefly. Color changes were then observed: retention of the blue color indicated inhibition of bacterial growth, whereas a pink or colorless appearance indicated bacterial proliferation. The minimum inhibitory concentration (MIC) was defined as the lowest concentration at which no color change was observed.

For determination of the minimum bactericidal concentration (MBC), samples were taken from wells showing no visible growth in the MIC assay and inoculated onto Mueller–Hinton Agar (MHA). The plates were incubated at 37 °C for 18–24 h and subsequently examined for colony formation. The lowest concentration at which no colony growth was observed was considered the MBC value.

In the agar well diffusion method, bacterial suspensions adjusted to a 0.5 McFarland turbidity standard were uniformly spread onto the surface of MHA plates. Following inoculation, plates were allowed to stand for approximately 5 min to ensure proper absorption. Wells of appropriate diameter were then aseptically created in the agar. The test compounds were prepared at a concentration of 0.5 mg/mL, and 100 μL of each was introduced into the wells. The plates were incubated at 37 °C, and antibacterial activity was evaluated by measuring the diameters of the inhibition zones in millimeters after incubation.

#### 2.2.3. Bacterial Morphology

The morphological alterations induced by compound **1c** on MRSA cells were examined using scanning electron microscopy (SEM). In the experimental setup, 1 mL of bacterial suspension with an approximate density of 1 × 10^6^ CFU/mL was transferred into 12-well plates. Subsequently, 1 mL of the test compound prepared at 2 × MIC was added, and the plates were incubated at 37 °C for 24 h. Wells containing only culture medium were used as the control group.

Following incubation, the cell samples were collected and fixed in 3% glutaraldehyde solution at 4 °C overnight. After fixation, the samples were subjected to a graded ethanol series for dehydration to remove water content. Upon completion of the drying process, the sample surfaces were coated with a thin layer of gold to enhance conductivity. Finally, the surface morphology of the cells was examined under SEM, and the observed structural changes were recorded [24].

#### 2.2.4. Antibiofilm Activity

In this study, the crystal violet assay was employed to evaluate the effects of compounds **1a**–**1e** on MRSA biofilm formation. Bacterial strains were incubated in an appropriate growth medium for 18–24 h to obtain fresh cultures. The bacterial suspension was adjusted to a 0.5 McFarland standard and further diluted for use in biofilm assays. A volume of 200 µL of the prepared bacterial suspension was dispensed into sterile 96-well microplates and incubated at 37 °C for 24 h to allow biofilm formation.

Following incubation, planktonic bacteria were carefully removed, and compounds **1a**–**1e** at concentrations of 2 × MIC, 1 × MIC, and 1/2 × MIC were added to the wells. The plates were then re-incubated under the same conditions. After incubation, the wells were gently washed with PBS to remove non-adherent cells. Biofilms were fixed using methanol, followed by staining with 0.05% crystal violet solution. Excess stain was removed, and the plates were washed and allowed to dry completely.

Subsequently, 96% ethanol was added to solubilize the bound dye, and the absorbance of the resulting solution was measured at 570 nm using a microplate reader [25].

Antibiofilm rate (%)=(C−S) / C × 100%

where C = absorbance of the control (biofilm, no treatment), and S = absorbance of the test (biofilm and treatment)

#### 2.2.5. Gene Expression

The effect of the most active compound, **1c**, against MRSA—identified based on microdilution and agar well diffusion assays—was evaluated by analyzing the expression levels of target genes *icaA*, *dltA*, *dltB*, *sarA*, *norA*, and *norB*, using *16S rRNA* as the reference gene [26].

Following 24 h treatment of bacterial cultures with the selected compound, total RNA was isolated using TRIzol reagent (Nucleogene Tri-Reagent NGE-023, Kartal/İstanbul, Türkiye) according to the manufacturer’s protocol. The purity and concentration of the extracted RNA samples were determined using a NanoDrop spectrophotometer (DeNovix DS-11, Wilmington, DE, USA). RNA concentrations were standardized to 200 ng/µL prior to downstream applications.

Subsequently, cDNA synthesis was performed from the isolated RNA samples using the Roche Transcriptor First Strand cDNA Synthesis Kit (Roche, Basel, Switzerland). The synthesis protocol was carried out at 25 °C for 10 min, followed by 55 °C for 30 min, and a final step at 85 °C for 5 min.

Quantitative real-time PCR (qPCR) analysis was conducted using LightCycler^®^ FastStart DNA Master SYBR Green I (Roche, Basel, Switzerland). Amplification reactions were performed on an LC96 instrument (Roche, Basel, Switzerland ) under the following conditions: initial denaturation at 95 °C for 5 min, followed by 40 cycles of 95 °C for 5 s and primer-specific annealing temperatures for 30 s.

Relative gene expression levels were calculated using the 2^−ΔΔCt^ method.

#### 2.2.6. Antioxidant Assays 

The antioxidant activities of compounds **1a**–**1e** were evaluated using the DPPH radical scavenging assay. A freshly prepared 0.10 mM DPPH solution (in methanol) was protected from light and mixed with the test compounds at different concentrations (25–100 µg/mL). After vortexing, the reaction mixtures were incubated at room temperature for 30 min, and the absorbance was measured at 517 nm using a UV–Vis spectrophotometer. A sample without the test compound was used as the control, while butylated hydroxytoluene (BHT; 5–80 µg/mL) served as the standard antioxidant. Radical scavenging activity was calculated as a percentage relative to the control [27].

Similarly, ABTS radical scavenging activity was assessed using a spectrophotometric method. The ABTS radical solution was generated by reacting 7 mM ABTS with 140 mM potassium persulfate and allowing the mixture to stand in the dark at room temperature for 16 h. The resulting ABTS solution was then mixed with compounds **1a**–**1e** at varying concentrations (25–100 µg/mL), followed by vortexing and incubation for 30 min. Absorbance was recorded at 734 nm. BHT was again used as the reference standard, and antioxidant activity was calculated relative to the control solution [27].

#### 2.2.7. Molecular Docking

In molecular docking studies, the interactions of compound **1c**—the most active compound identified in this study and whose chemical structure was drawn using the ChemDraw software v15.0—were comprehensively evaluated against selected target proteins (1MWT, 3ZG5, 1JIJ, and 2Y2M) that play critical roles in the physiology and pathogenicity of *Staphylococcus aureus*. These targets were chosen based on their involvement in essential biological processes such as cell wall biosynthesis, metabolic pathways, and bacterial survival mechanisms.

All molecular modeling and docking simulations were performed using the Maestro interface of the Schrödinger software suite. The three-dimensional crystal structures of the target proteins were retrieved from the Protein Data Bank (PDB) and prepared using the Protein Preparation Wizard module, which included the addition of missing hydrogen atoms, assignment of bond orders, optimization of hydrogen bonding networks, and energy minimization under appropriate force field parameters.

The ligand structure (compound **1c**) was prepared using the LigPrep module, where possible ionization states, tautomeric forms, and stereoisomers were generated, followed by geometry optimization. Docking calculations were carried out using the Glide module in standard precision (SP) and/or extra precision (XP) modes to predict the binding orientation and affinity of the ligand within the active sites of the target proteins. Key ligand–protein interactions, including hydrogen bonding, hydrophobic contacts, π–π stacking, and electrostatic interactions, were analyzed in detail.

To further validate the docking results and assess the stability of the ligand–protein complexes, binding free energy calculations were performed using the MM-GBSA (Molecular Mechanics Generalized Born Surface Area) approach. The ΔG_bind values provided insights into the thermodynamic favorability of the interactions, allowing a more reliable estimation of binding strength. Overall, these computational analyses were conducted to elucidate the potential molecular mechanisms underlying the antibacterial activity of compound **1c** and to support the experimental findings at the molecular level [28].

## 3. Results

### 3.1. Spectroscopic Analysis of the Compounds

The ^1^H NMR spectra of compounds **1a**–**1e** were analyzed to confirm their structural features (Supplementary Materials). Signals corresponding to the methyl (-CH_3_) group were observed at 2.23 ppm, integrating for three protons in all compounds. The methylene (-CH_2_-) bridges located on both sides of the benzimidazole core appeared as two singlets around 5.5 ppm, integrating for a total of four protons. In compound **1e**, an additional methoxy (-OCH_3_) proton signal was observed at 3.81 ppm, integrating for three protons. A singlet at 13.06 ppm, integrating for one proton, was assigned to the -OH proton of the carboxylic acid group in compound **1d**. Aromatic protons were observed in the region of 7.04–8.00 ppm. All integration values were consistent with the proposed structures.

The ^13^C NMR spectra of the compounds confirmed their proposed structures (Supplementary Materials). The signal corresponding to the para-positioned methyl (-CH_3_) carbon was observed at approximately 21.1 ppm for all compounds (**1a**–**1e**). The methylene (-CH_2_-) carbons on both sides of the benzimidazole core appeared at around 49.4 ppm. In compound **1e**, the methoxy (-OCH_3_) carbon resonated at 52.7 ppm. Aromatic carbons were observed in the range of 110.0–151.7 ppm. Additionally, the carbonyl (C=O) carbons of compounds **1d** and **1e** appeared at 167.2 and 166.2 ppm, respectively. All carbon signals were consistent with the proposed structures. The FT-IR spectra showed a weak aromatic C–H stretching band around 3000 cm^−1^. The symmetric and asymmetric stretching vibrations of the methylene (-CH_2_-) group were detected around 2900 cm^−1^. A weak absorption band attributed to the benzimidazole C=N stretching vibration was observed near 1615 cm^−1^. The presence of characteristic absorption bands for the major functional groups within their expected frequency ranges confirms that the FT-IR spectra are in agreement with the proposed structures of the synthesized compounds.

### 3.2. Evaluation of Antimicrobial Activity

**Figure 2:**
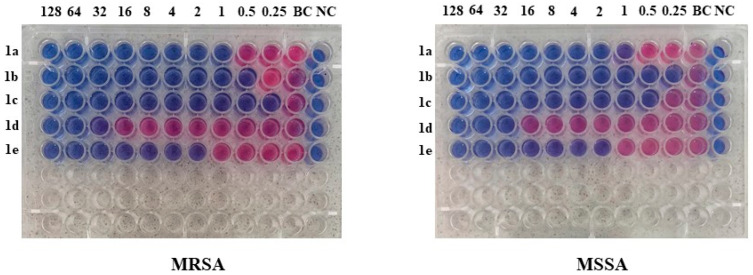
Resazurin-based MIC assay.

In this study, the minimum inhibitory concentration (MIC) values of compounds **1a**–**1e** against MRSA and MSSA strains were determined using the resazurin assay (Figure 2). The findings revealed that the tested compounds exhibited varying levels of inhibitory activity against both MRSA and MSSA strains.

For the MRSA strain, the MIC values were determined as 1 µg/mL for **1a**, 0.5 µg/mL for **1b**, 0.25 µg/mL for **1c**, 32 µg/mL for **1d**, and 2 µg/mL for **1e**. For the MSSA strain, the MIC values were found to be 2 µg/mL for **1a**, 0.25 µg/mL for **1b**, 0.5 µg/mL for **1c**, 32 µg/mL for **1d**, and 2 µg/mL for **1e** (Table 1).

For comparison, the MIC value of the reference antibiotic vancomycin was determined as 1 µg/mL for MSSA and 2 µg/mL for MRSA. The results demonstrated that compounds **1a**, **1b**, and **1c** exhibited stronger antibacterial activity against MRSA than vancomycin, as indicated by their lower MIC values. Similarly, compounds **1b** and **1c** showed greater potency against MSSA compared to vancomycin.

The MBC values ranged between 0.5 and 8 µg/mL for both MSSA and MRSA strains (Table 1).

Overall, compounds **1b** and **1c** displayed remarkable antibacterial activity against both strains with notably low MIC values. In particular, compound **1c** was the most effective against MRSA, while compound **1b** showed the highest activity against MSSA. In contrast, compound **1d** exhibited the weakest antibacterial activity against both bacterial strains.

**Figure 3:**
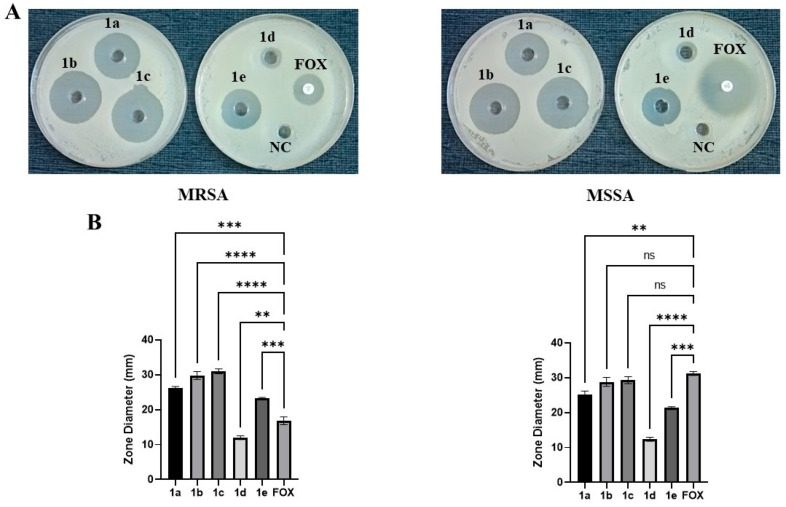
Inhibition zone diameters (mm) of compounds **1a**–**1e** determined by the agar well diffusion method: (**A**) overall inhibition zones, (**B**) antibacterial activity against MRSA. Data are presented as mean ± SD (n = 3). ns indicates not significant (*p* ≥ 0.05); ** *p* < 0.01, *** *p* < 0.001, and **** *p* < 0.0001 were considered statistically significant.

The results of the agar well diffusion assay demonstrated that the tested compounds exhibited varying levels of antibacterial activity against both MRSA and MSSA strains (Figure 3).

For the MRSA strain, the inhibition zone diameters for compounds **1a**, **1b**, **1c**, **1d**, and **1e** were measured as 26.1 ± 0.4, 29.8 ± 0.8, 31.0 ± 0.5, 12.0 ± 0.4, and 23.2 ± 0.2 mm, respectively. In the MSSA strain, the inhibition zone diameters were determined as 25.2 ± 0.7, 28.8 ± 0.9, 29.0 ± 0.7, 12.4 ± 0.4, and 21.3 ± 0.3 mm for compounds **1a**, **1b**, **1c**, **1d**, and **1e**, respectively.

The inhibition zone diameter of the reference antibiotic cefoxitin (FOX) was 16.8 ± 0.8 mm for MRSA and 31.2 ± 0.4 mm for MSSA. The findings revealed that compounds **1a**, **1b**, and **1c** exhibited stronger antibacterial activity against MRSA than FOX, as evidenced by their larger inhibition zones. In the MSSA strain, compound **1c** showed activity comparable to FOX, while compounds **1a** and **1b**, although demonstrating notable antibacterial effects, exhibited smaller inhibition zones compared to FOX.

Overall, compounds **1b** and particularly **1c** displayed remarkable antibacterial efficacy against both MRSA and MSSA strains, highlighting their potential as strong antimicrobial candidates.

### 3.3. Evaluation of Bacterial Morphology

**Figure 4:**
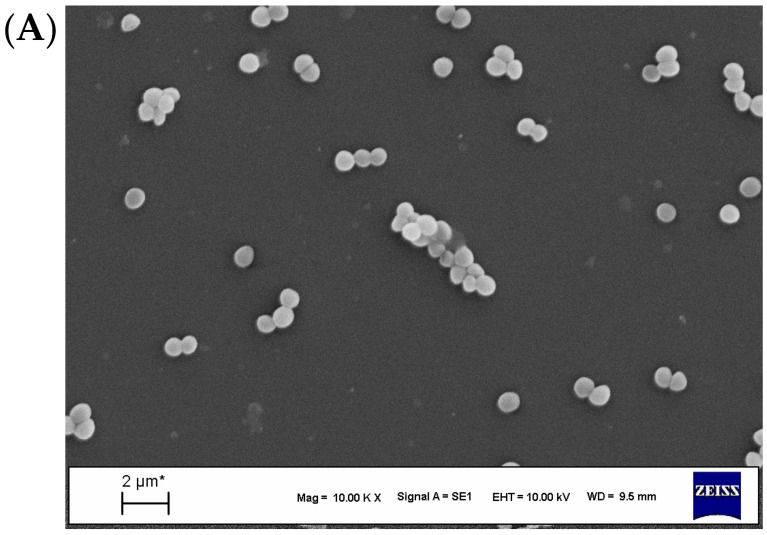

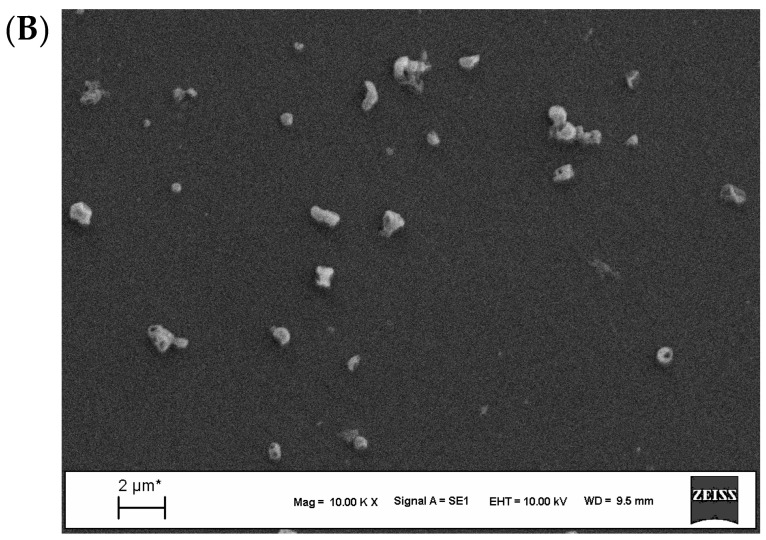
SEM images of MRSA: (**A**) control (untreated) group and (**B**) **1c**-treated group.

SEM analysis revealed that compound **1c** at 2 × MIC induced significant structural alterations in MRSA cell morphology. In the control group, MRSA cells exhibited smooth surfaces, a typical spherical morphology, and characteristic grape-like clustering. In contrast, in the group treated with compound **1c**, disruption of cell surface integrity, deformation of the cell wall, and marked alterations in bacterial cell morphology were observed (Figure 4).

### 3.4. Evaluation of Antibiofilm Activities

**Figure 5:**
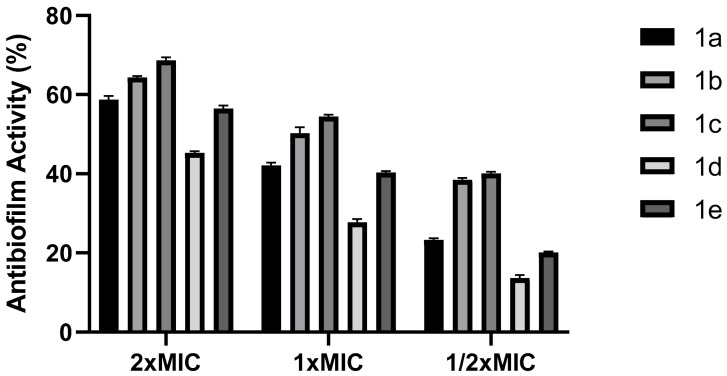
Antibiofilm activity of compound **1a**–**1e** against MRSA strain.

The effects of compounds **1a**–**1e** at different concentrations (2 × MIC, 1 × MIC, and 1/2 × MIC) on preformed MRSA biofilms were evaluated, revealing that all compounds exhibited dose-dependent inhibitory effects on biofilm biomass (Figure 5). The highest antibiofilm activity was observed at the 2 × MIC, where all compounds effectively suppressed preformed MRSA biofilms. At this concentration, the highest inhibition rate was recorded for compound **1c** (68.6 ± 0.6%), followed by **1b** (64.3 ± 0.4%), **1a** (58.7 ± 0.7%), **1e** (56.5 ± 0.6%), and **1d** (45.3 ± 0.3%).

At 1 × MIC, all compounds continued to exhibit significant inhibitory effects on preformed MRSA biofilms; however, the antibiofilm activity was lower compared to that observed at 2 × MIC. Similarly, at 1/2 × MIC, all tested compounds maintained inhibitory effects, although at more limited levels. At this concentration, the highest biofilm inhibition was again observed for compound **1c** (40.1 ± 0.3%), followed by **1b** (38.4 ± 0.4%), **1a** (23.3 ± 0.3%), **1e** (20.1 ± 0.2%), and **1d** (13.6 ± 0.6%).

Overall, among the tested compounds, **1c** demonstrated the strongest antibiofilm activity across all concentrations, consistently exhibiting the highest inhibition rates against preformed MRSA biofilms.

### 3.5. Gene Expression Analysis

To elucidate the molecular basis of the antibacterial and antibiofilm effects of the most active compound, **1c**, gene expression analyses were performed. The target genes included *icaA*, *dltA*, *dltB*, *mepA*, *norA*, and *norB*. The results demonstrated that the Log2 fold change values of all analyzed genes were downregulated compared to the control group.

**Figure 6:**
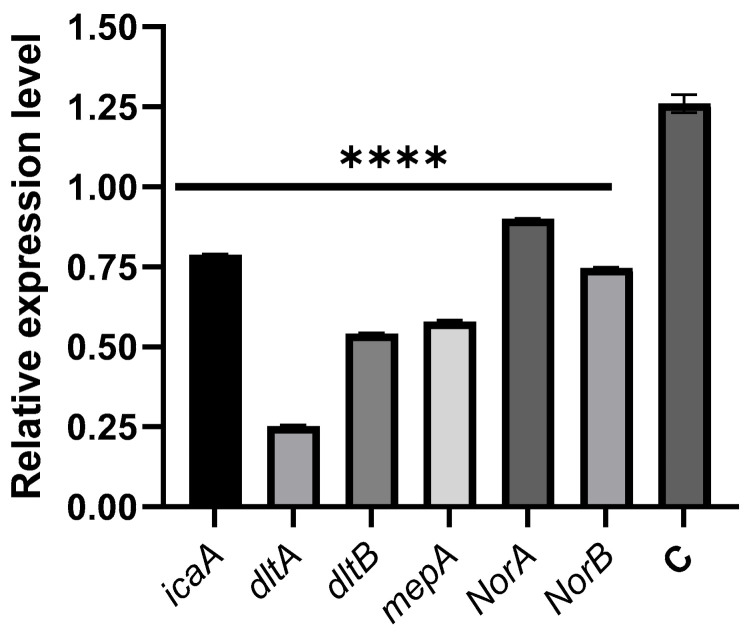
Analysis of gene expression in MRSA. **** *p* < 0.0001 were considered statistically significant.

The most pronounced decrease in gene expression was observed for *dltA*, with a Log2 fold change value of −2.324. This was followed by reductions in *dltB* (−1.219) and *mepA* (−1.125). The biofilm-associated *icaA* gene exhibited a more moderate downregulation, with a Log2 fold change value of −0.681. Similarly, efflux pump-related genes *norA* and *norB* also showed decreased expression levels, with Log2 fold change values of −0.487 and −0.758, respectively (Figure 6).

### 3.6. Antioxidant Activity

**Figure 7:**
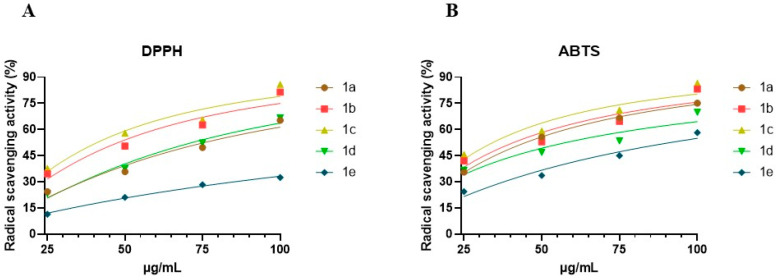
Antioxidant activity of **1a**–**1e** compounds. (**A**) DPPH, and (**B**) ABTS.

The antioxidant potential of compounds **1a**–**1e** was investigated using DPPH and ABTS radical scavenging assays at different concentrations. The results revealed notable differences in antioxidant performance among the tested compounds (Figure 7).

In the DPPH assay, at the lowest concentration (25 µg/mL), the radical scavenging activities of the compounds ranged between 11.4% and 37.8%. As the concentration increased, antioxidant activity also increased. At the highest concentration (100 µg/mL), compound **1c** exhibited the highest radical scavenging activity at 85.8%, followed by compound **1b** with 81.4%.

In the ABTS assay, the tested compounds showed radical scavenging activities ranging from 24.4% to 45.6% at the lowest concentration. At the highest concentration, compound **1c** demonstrated 89.4% antioxidant activity, followed by compound **1b** with 83.2%. Also, at the highest tested concentration of BHT (80 µg/mL), DPPH and ABTS radical scavenging activities were determined to be 82.3% and 91.2%, respectively.

Overall, the evaluation indicated that compounds **1b** and **1c** exhibited stronger antioxidant properties in the tested systems, as reflected by their higher antioxidant activity percentages.

### 3.7. Molecular Docking

To support the experimental findings, molecular docking studies were conducted for compound **1c**, identified as the most active among the synthesized compounds, using four selected target proteins. The calculated docking scores were found to be relatively similar across all targets. Among them, the strongest interaction was observed with the 2Y2M protein, with a docking score of −5.322 kcal/mol, whereas the weakest interaction was recorded for 1MWT with a docking score of −5.137 kcal/mol (Table 2).

**Figure 8:**
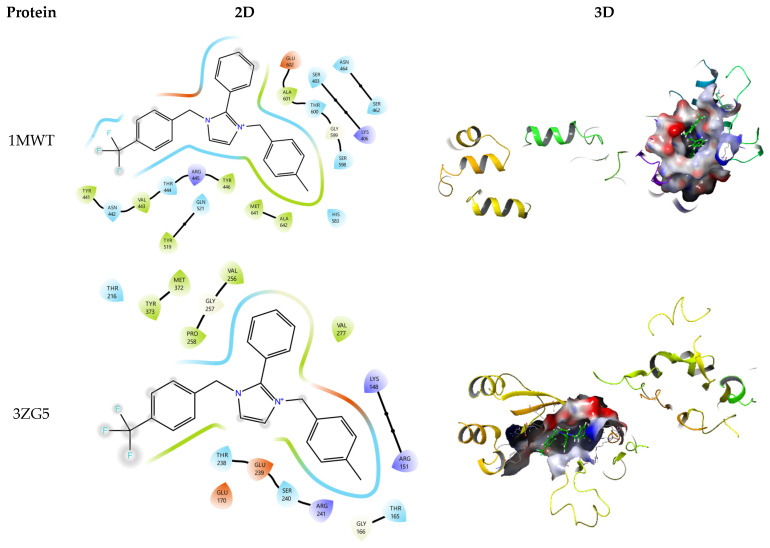

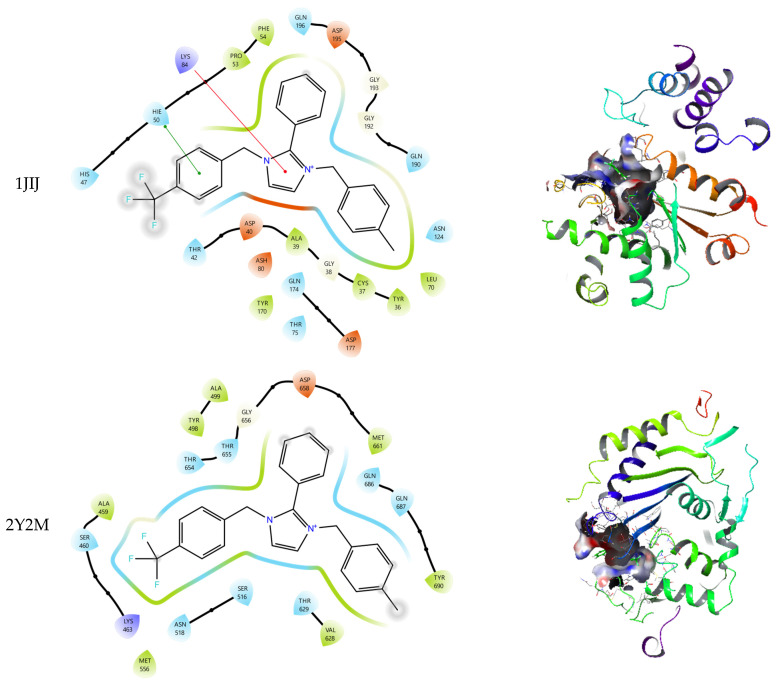
2D and 3D representations of the binding interactions of compound **1c** in the active sites of selected bacterial target proteins.

In addition to docking scores, MM-GBSA binding free energy values were evaluated, as they provide a more reliable estimation of binding stability. According to the obtained results, the binding free energies were calculated to be −48 kcal/mol or lower, indicating favorable and stable ligand–protein interactions. Molecular docking analysis demonstrated that compound **1c** interacts with the 1JIJ protein through a π–π stacking interaction with HIE50 and a π–cation interaction with LYS84. Such non-covalent interactions are known to enhance ligand–protein complex stability and are therefore likely to contribute significantly to the favorable binding orientation and affinity of compound **1c** within the active site (Figure 8).

## 4. Discussion

The emergence and global dissemination of MRSA pose a serious public health concern due to its ability to cause infections that are difficult to treat and of high clinical significance [29]. Therefore, the development of novel antimicrobial agents effective against MRSA has become one of the primary priorities of modern medicine [30]. Compounds containing the benzimidazole core are among the most important heterocyclic structures widely used in medicinal chemistry and drug design, and their novel biological applications are being extensively investigated. Due to their reported anticancer [31], antibacterial [32,33], antibiofilm [34], antiviral [35], and antioxidant activities [36], benzimidazole derivatives are considered valuable pharmacophores in the development of new drug candidates.

In our previous study, benzimidazolium–chalcone derivatives exhibited MIC values ranging from 2 to 64 μg/mL against MSSA and MRSA strains. Additionally, inhibition zone diameters of 11–21 mm were observed for both strains [26]. In the literature, Chen et al. reported that a fluorinated benzimidazole derivative showed an MIC value of 4 μg/mL against MRSA [37]. Tessier et al. determined MIC values ranging from 1.25 to 5 μg/mL for benzimidazolium salts against MRSA [38]. Zhang et al. reported that the most effective MIC value among benzimidazole derivatives against *S. aureus* was 2 μg/mL [39]. In contrast, Alasmary et al. reported significantly higher MIC values (32 to >512 μg/mL) for benzimidazolium derivatives against MRSA, with inhibition zones ranging from 9 to 15 mm [40].

In the present study, MIC results demonstrated that compounds **1a**–**1e** possess notable antibacterial potential against both bacterial strains. Agar well diffusion results further supported these findings, particularly highlighting compound **1c** as the most potent agent against MRSA, producing the largest inhibition zone. Similarly, MIC data indicated that compound **1c** exhibited the strongest inhibitory effect against MRSA, while compound **1b** showed the lowest MIC value and highest antibacterial activity against MSSA. In conclusion, compounds **1a**, **1b**, and **1c** exhibited higher antibacterial activity against MRSA compared to FOX. This enhanced activity may be associated with the cationic benzimidazolium core, which can interact electrostatically with negatively charged bacterial membranes, causing membrane destabilization and increased permeability. In addition, the lipophilic substituents attached to the benzimidazolium scaffold may improve penetration through the bacterial cell wall and facilitate interactions with intracellular targets [41,42]. In addition, negatively charged extracellular DNA, which is an important structural component of the biofilm matrix, may also play a role in the observed antibacterial activity. Due to their cationic structure, benzimidazolium salts are capable of interacting with DNA through electrostatic interactions, thereby promoting biofilm penetration and destabilization [43]. These findings suggest that the investigated compounds may serve as promising therapeutic agents, particularly for the treatment of resistant staphylococcal infections, and provide valuable leads for the development of next-generation antimicrobials.

The ability of bacterial pathogens to form biofilms is considered one of the major causes of morbidity and mortality in chronic and hospital-acquired infections [44]. In particular, *Staphylococcus aureus* is a prominent pathogen due to its capacity to adhere to surfaces and develop robust biofilm structures [45]. Biofilms are defined as structured microbial communities embedded in a self-produced extracellular polymeric substance (EPS) matrix, which acts as a protective barrier against environmental stress [46]. This structure enables bacterial cells within the biofilm to evade host immune responses and develop increased resistance to antibiotics. Consequently, biofilm-associated infections are difficult to treat, prone to chronicity, and associated with high recurrence rates. Conventional antibiotic therapies are often insufficient against these structures, highlighting the need for alternative therapeutic strategies.

Previous studies have shown that the antibiofilm activity of benzimidazolium salts against MRSA is associated with their ability to disrupt the biofilm matrix and bacterial cell membranes [38]. Kong et al. investigated the antibiofilm activity of the benzimidazole derivative UM-C162 against MRSA and reported that it inhibited biofilm formation in a dose-dependent manner [34]. Furthermore, antibiofilm activity of benzimidazolium derivatives against MRSA has also been reported in our previous work [47]. In the present study, compounds **1a**–**1e** exhibited concentration-dependent antibiofilm activity, with compound **1c** demonstrating the most pronounced effect against MRSA biofilms.

Gene expression analysis indicated that even short-term exposure to compound **1c** resulted in significant transcriptional suppression of both biofilm-associated and efflux pump-related genes in MRSA. Notably, the downregulation of *dltA* (−2.324) and *dltB* (−1.219) was particularly prominent. The *dlt* operon plays a critical role in regulating cell surface charge through D-alanylation of teichoic acids in Gram-positive bacteria, and its suppression has been associated with increased β-lactam susceptibility and reduced biofilm formation in MRSA [48]. Additionally, the decreased expression of *icaA* (−0.681) suggests interference with polysaccharide intercellular adhesion and biofilm matrix formation.

Regarding efflux pump genes, the downregulation of *mepA* (−1.125), *norA* (−0.487), and *norB* (−0.758) indicates that compound **1c** may suppress membrane transport systems associated with multidrug resistance in MRSA. Chen et al. evaluated the antibacterial and antibiofilm effects of the benzimidazole derivative 2-(3,5-bis(trifluoromethyl)phenyl)-4-nitro-6-(trifluoromethyl)-1H-benzo[d]imidazol-1-ol (TFBZ) on MRSA and performed gene expression analysis using qRT-PCR [37]. Consistent with our findings, they reported a decrease in the expression of *icaA* and *icaD* genes [37].

In *S. aureus*, efflux pump genes such as *norA*, *norB*, and *mepA* are associated with the extrusion of antibiotics from the cell and the development of resistance to various antimicrobial agents [49,50]. Consequently, compound **1c** can disrupt bacterial cell membrane permeability and integrity, while also suppressing efflux pump gene expression, thereby weakening active efflux mechanisms. These combined effects contribute to the effective eradication of MRSA biofilms [51].

Importantly, unlike many previously reported studies that primarily focus on phenotypic antimicrobial screening, the present work additionally investigates these key genetic determinants, thereby providing a more comprehensive understanding of the underlying molecular mechanisms. This mechanistic approach represents a significant advantage of the current study, as it allows correlation of antibacterial activity with specific pathways involved in MRSA pathogenicity and resistance.

Excessive production and accumulation of free radicals beyond physiological limits disrupt oxidative balance, leading to the development of oxidative stress. Reactive oxygen species (ROS), which can cause damage to proteins, lipids, and nucleic acids at the cellular level, must be tightly regulated to maintain biological homeostasis. Therefore, the development of novel therapeutic strategies aimed at limiting oxidative stress-induced cellular damage has gained increasing importance in recent years. In this context, supporting biological systems with antioxidant compounds is considered one of the most effective protective approaches. Recent studies have demonstrated that not only natural compounds but also synthetic heterocyclic structures can exhibit strong antioxidant properties. In particular, newly developed indole- and benzimidazole-based derivatives have been reported to possess remarkable antioxidant potential due to their free radical scavenging activities [52,53].

Ouasif et al. reported that 2-mercaptobenzimidazole derivatives exhibit significant DPPH radical scavenging capacity, demonstrating notable antioxidant potential [54]. Similarly, Argirova et al. investigated the antioxidant activities of novel 1H-benzimidazole-2-yl hydrazone derivatives bearing hydroxyphenyl and methoxyphenyl groups using ABTS and DPPH assays, and found that some derivatives exhibited higher radical scavenging activity than standard reference antioxidants [55]. In another study, Albayrak et al. reported that synthesized benzimidazolium salts showed moderate inhibitory effects on the DPPH radical (29.53–39.75%) [56].

In the present study, evaluation of the antioxidant activities of compounds **1a**–**1e** revealed that compound **1c** exhibited very high antioxidant activity in both DPPH and ABTS radical scavenging assays. The combined assessment of DPPH and ABTS results suggests that benzimidazolium derivatives possess not only antimicrobial properties but also potential biological activity in oxidative stress-related processes. This dual functionality indicates that these compounds may serve as multifunctional pharmacological candidates, particularly in reducing oxidative damage associated with infection and inflammation. Therefore, the antioxidant properties of benzimidazolium derivatives can be considered a significant biological advantage that enhances their therapeutic value.

## 5. Conclusions

In the present study, five benzimidazolium salts with different substituents were successfully synthesized and structurally characterized by NMR and FTIR analyses. The biological evaluation demonstrated that these compounds possess significant antibacterial activity against both MRSA and MSSA strains. Among them, compound **1c** emerged as the most potent derivative, exhibiting the lowest MIC values and the largest inhibition zones, indicating strong antibacterial efficacy.

SEM analysis further confirmed that compound **1c** induces pronounced morphological alterations in bacterial cells, suggesting disruption of cell membrane integrity as a possible mechanism of action. In addition, gene expression studies revealed that compound **1c** significantly downregulated key virulence- and resistance-associated genes, including *icaA*, *dltA*, *dltB*, *sarA*, *norA*, and *norB*, indicating its ability to interfere with biofilm formation and efflux-mediated resistance mechanisms. Consistently, antibiofilm assays showed that all compounds exhibited inhibitory activity, with compound **1c** displaying the highest antibiofilm effect.

Molecular docking studies supported the experimental findings, demonstrating favorable interactions of compound **1c** with selected target proteins (1MWT, 3ZG5, 1JIJ, and 2Y2M), with the strongest binding affinity observed for 2Y2M. These interactions, together with favorable binding free energy values, suggest stable ligand–protein complex formation at the molecular level.

Overall, the results indicate that benzimidazolium derivatives, particularly compound **1c**, exhibit potent antibacterial, antibiofilm, and molecular inhibitory activities. These findings highlight their potential as promising candidates for the development of new therapeutic agents against resistant *Staphylococcus aureus* infections. Further in vivo studies and detailed mechanistic investigations are warranted to fully explore their clinical applicability.

## Figures and Tables

**Table 1:** MIC and MBC values (µg/mL) of compounds **1a**–**1e** against MSSA and MRSA strains.

Bacterial Strain	1a		1b		1c		1d		1e		Vancomycin	
	MIC	MBC	MIC	MBC	MIC	MBC	MIC	MBC	MIC	MBC	MIC	MBC
MSSA	2	8	0.25	0.5	0.5	1	32	64	2	8	1	2
MRSA	1	2	0.5	1	0.25	0.5	32	64	2	8	2	4

**Table 2:** Molecular docking scores, Glide Emodel, Glide Gscore values and MM-GBSA (kcal/mol) of **1c**.

	Docking Score	Glide Emodel	Glide Gscore	MM-GBSA dG Bind
1MWT	−5.137	−55.846	−5.137	−54.13
3ZG5	−5.258	−52.998	−5.258	−52.33
1JIJ	−5.204	−58.685	−5.204	−48.23
2Y2M	−5.322	−54.851	−5.322	−55.07

## Data Availability

The original contributions presented in this study are included in the article and Supplementary Materials. Further inquiries can be directed to the corresponding author.

## Supplementary Materials

The following supporting information can be downloaded at: https://www.mdpi.com/article/10.3390/antibiotics15060567/s1, Figure S1: Compound **1a** ^1^H-NMR spectra, Figure S2: Compound **1a** ^13^C-NMR spectra, Figure S3: Compound **1b** ^1^H-NMR spectra, Figure S4: Compound **1b** ^13^C-NMR spectra, Figure S5: Compound **1c** ^1^H-NMR spectra, Figure S6: Compound **1c** ^13^C-NMR spectra, Figure S7: Compound **1d** ^1^H-NMR spectra, Figure S8: Compound **1d** ^13^C-NMR spectra, Figure S9: Compound **1e** ^1^H-NMR spectra, Figure S10: Compound **1e** ^13^C-NMR spectra, Figure S11: Compound **1a** FT-IR spectra, Figure S12: Compound **1b** FT-IR spectra, Figure S13: Compound **1c** FT-IR spectra, Figure S14: Compound **1d** FT-IR spectra, Figure S15: Compound **1e** FT-IR spectra.
