# Supplementary material for: Integrated In Vitro and In Silico Evaluation of Benzimidazolium Salts: Antioxidant Activity, Anti-MRSA/MSSA Effects, and Antibacterial Gene Expression Analysis
Source: Antibiotics (Basel). 2026 Jun 2;15(6):567. doi: 10.3390/antibiotics15060567 (PMC13296230; doi:10.3390/antibiotics15060567)
Supplement: Supplementary file 1 [file antibiotics-15-00567-s001.zip › antibiotics-4332211-supplementary.pdf]

Integrated *In Vitro* and *In Silico* Evaluation of Benzimidazolium Salts: Antioxidant Activity, Anti-MRSA/MSSA Effects, and Antibacterial Gene Expression Analysis

Başak Bedir [1], Hakan Ünver [2], Mehmet Çimentepe [3], Özge Öztürk Çimentepe [4], Akın Yiğin [5] and Metin Yildirim [6]*

[1] Department of Nutrition and Dietetics, Faculty of Health Science, Hakkari University Hakkâri, Turkey
[2] Department of Chemistry, Faculty of Science, Eskisehir Technical University, Eskisehir TR-26555, Türkiye
[3] Department of Pharmaceutical Microbiology, Faculty of Pharmacy, Harran University, Sanliurfa, Türkiye
[4] Department of Pharmacology, Faculty of Pharmacy, Harran University, Sanliurfa, Türkiye
[5] Department of Genetics, Faculty of Veterinary Medicine, Harran University, Sanliurfa TR-63200, Türkiye
[6] Department of Biochemistry, Faculty of Pharmacy, Cukurova University, Adana, Türkiye
* Correspondence: Metin Yildirim, meyildirim@cu.edu.tr

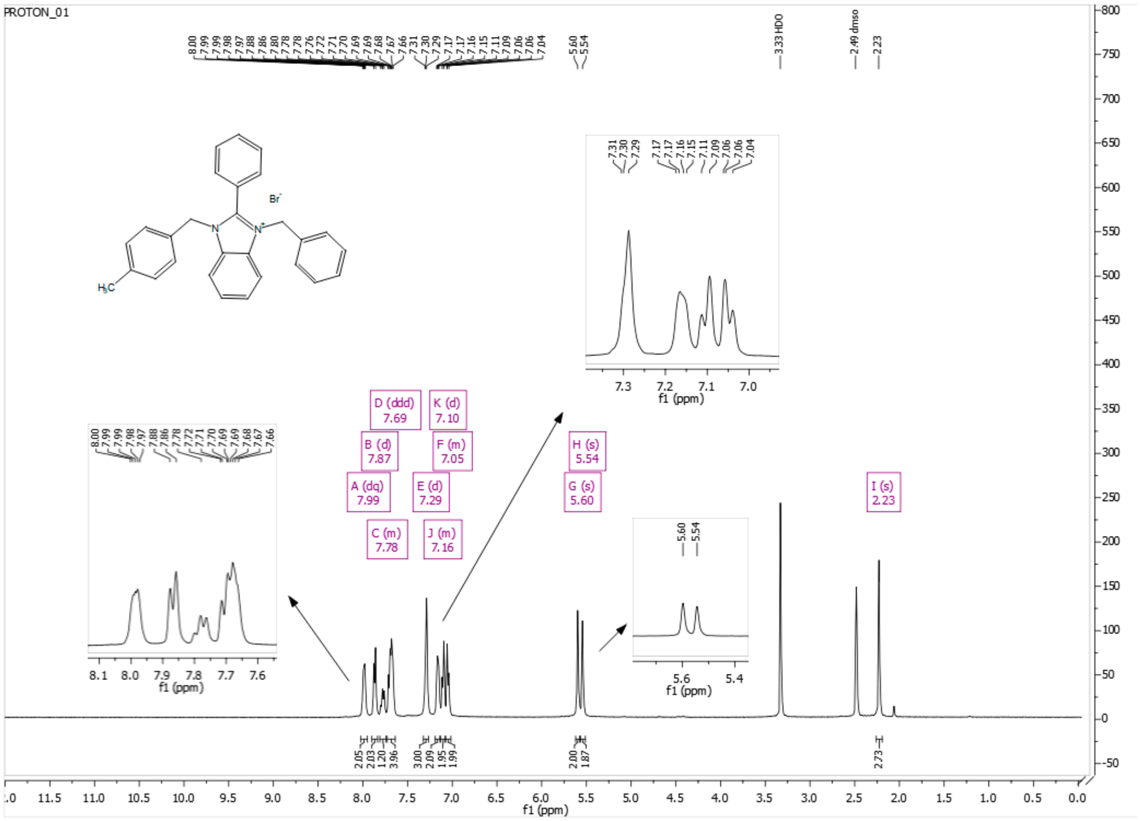

Figure S1: Compound 1a ^{1}H-NMR spectra

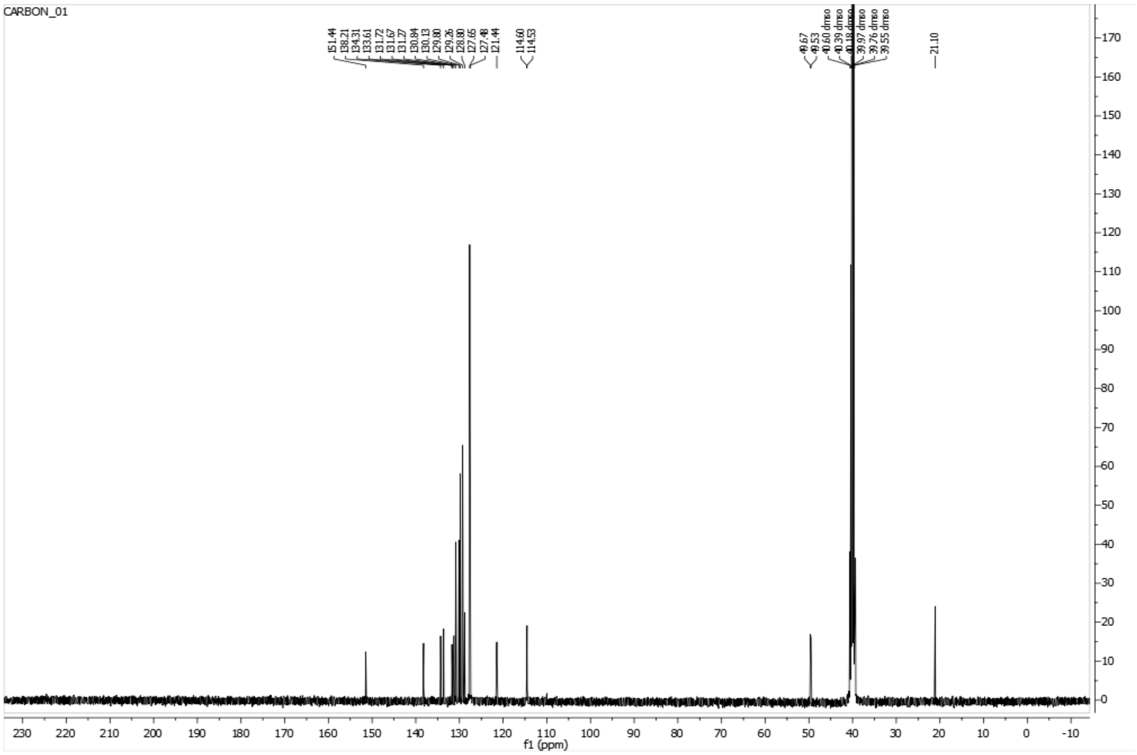

Figure S2: Compound 1a ^{13}C-NMR spectra

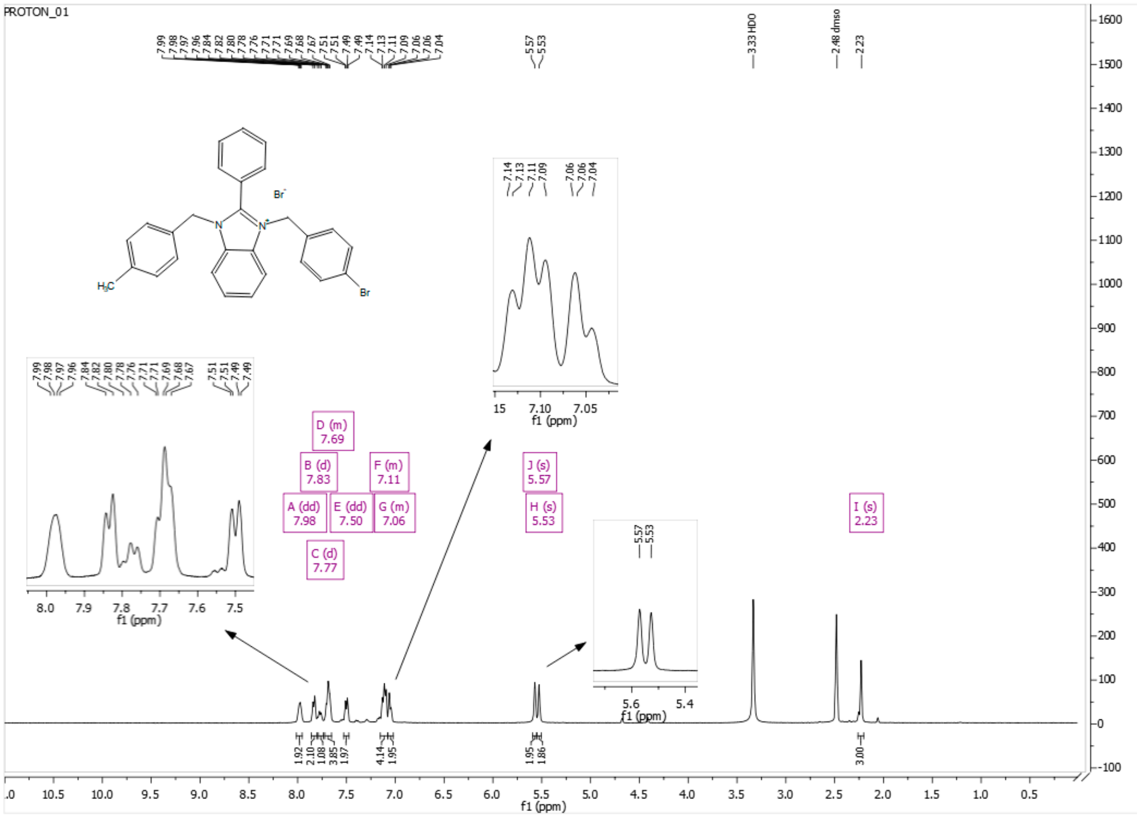

Figure S3: Compound 1b ^{1}H-NMR spectra

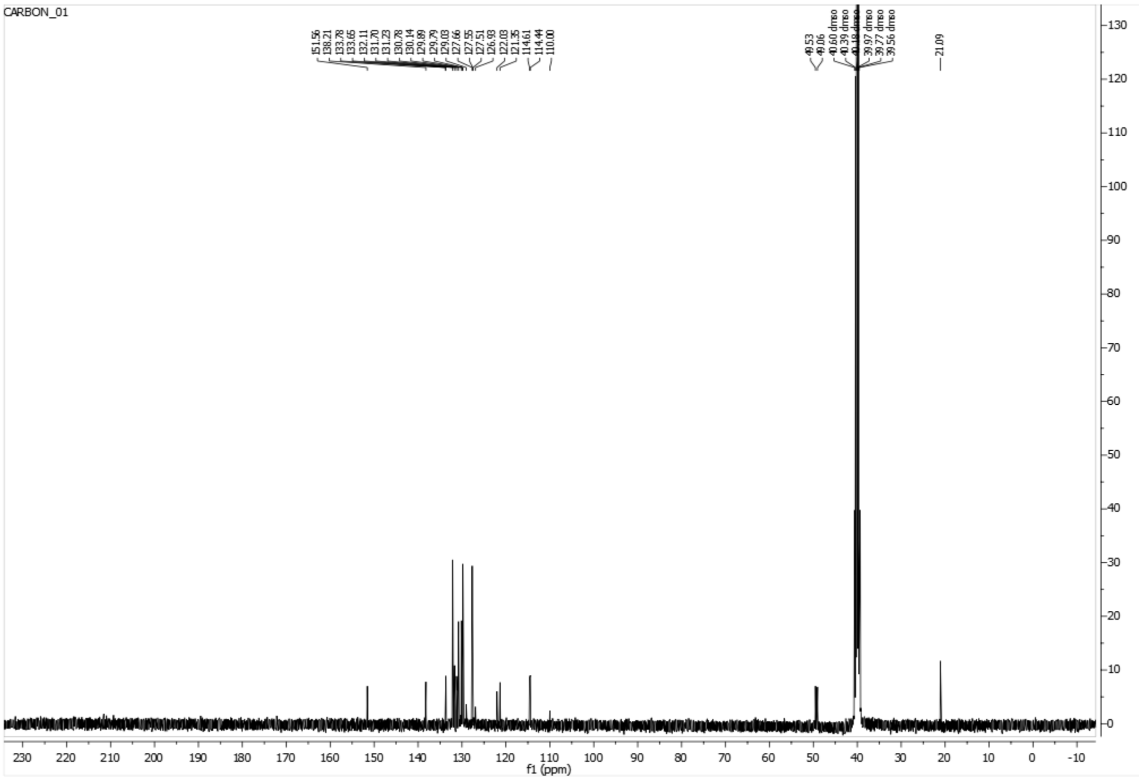

Figure S4: Compound 1b ^{13}C-NMR spectra

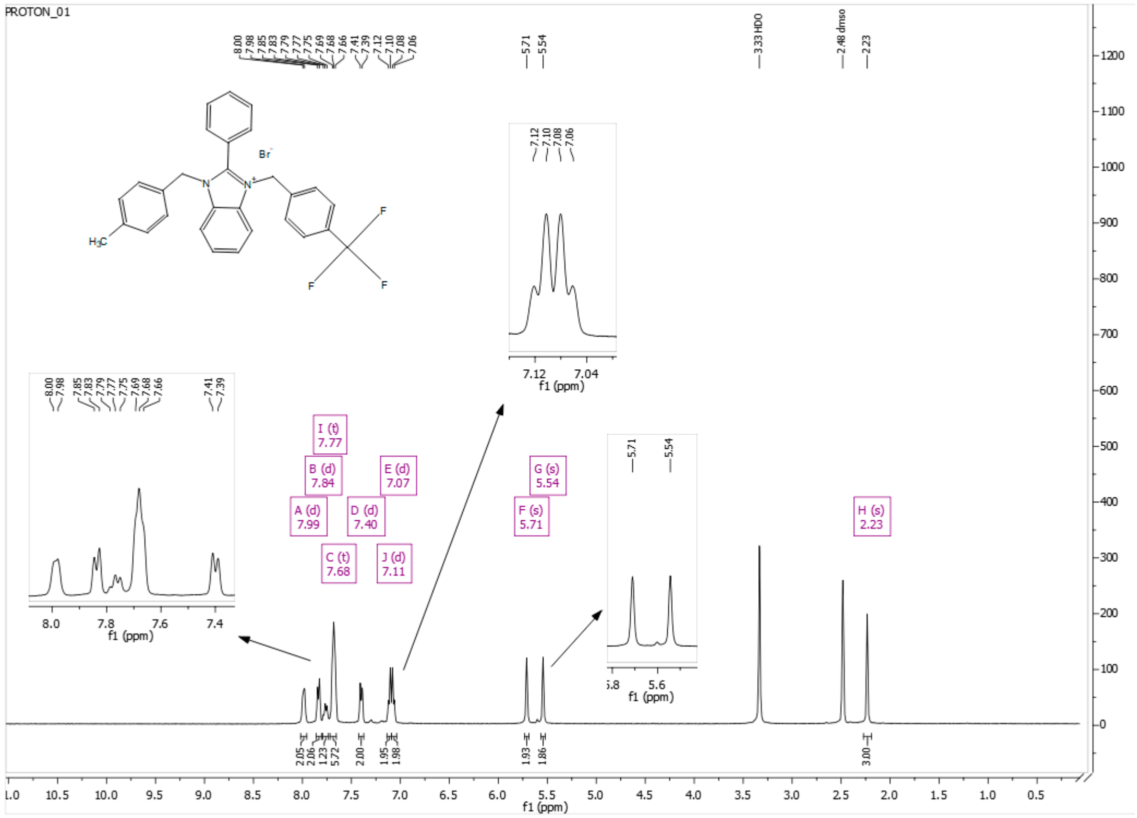

Figure S5: Compound 1c ^{1}H-NMR spectra

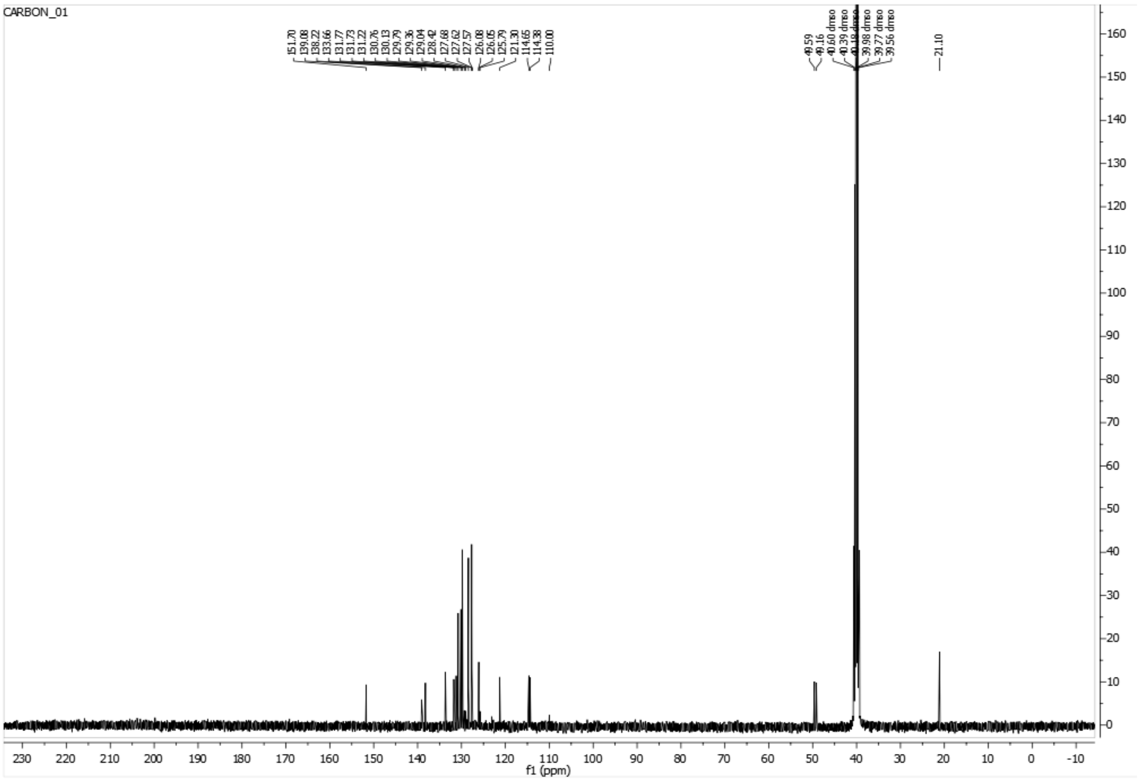

Figure S6: Compound 1c ^{13}C-NMR spectra

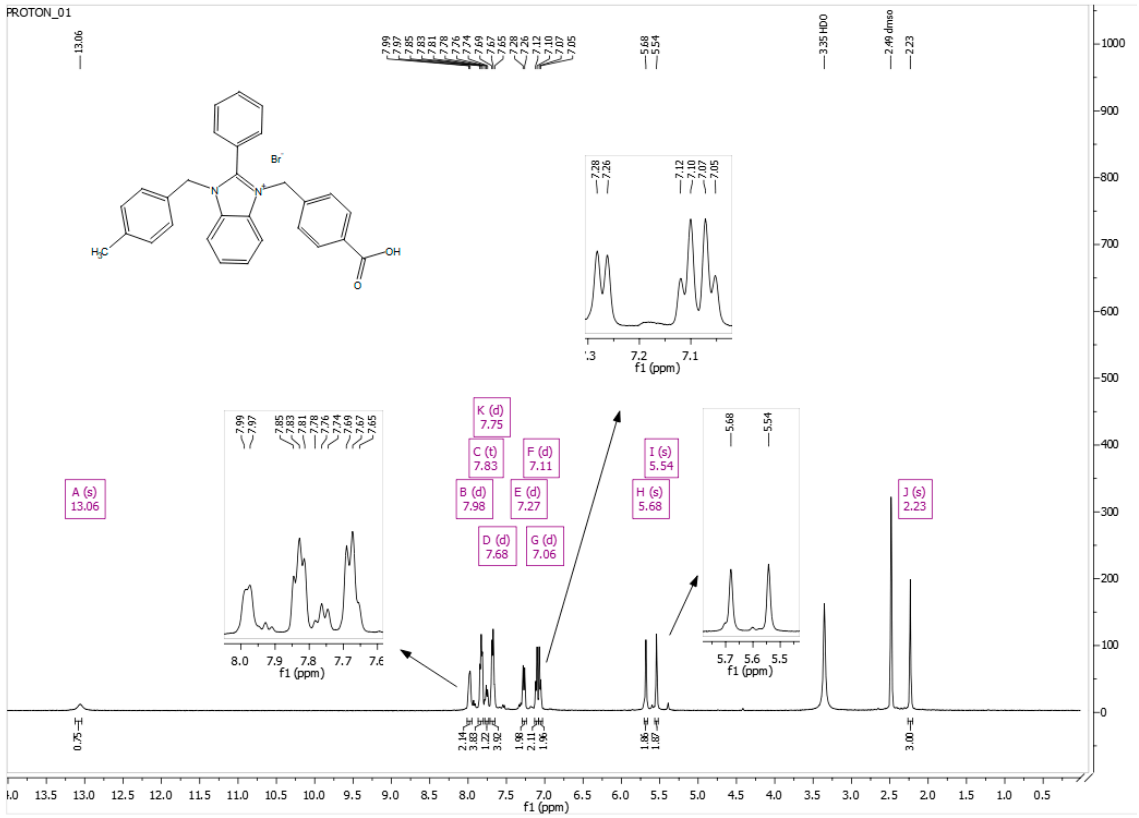

Figure S7: Compound 1d ^{1}H-NMR spectra

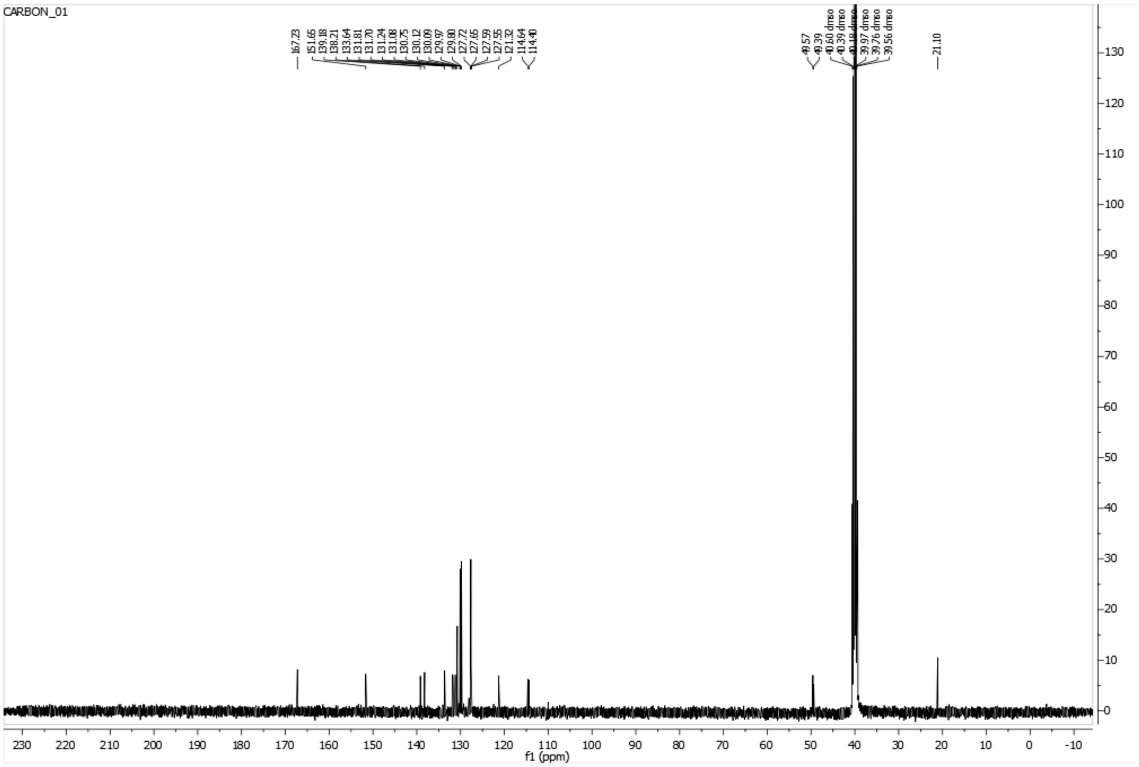

Figure S8: Compound 1d ^{13}C-NMR spectra

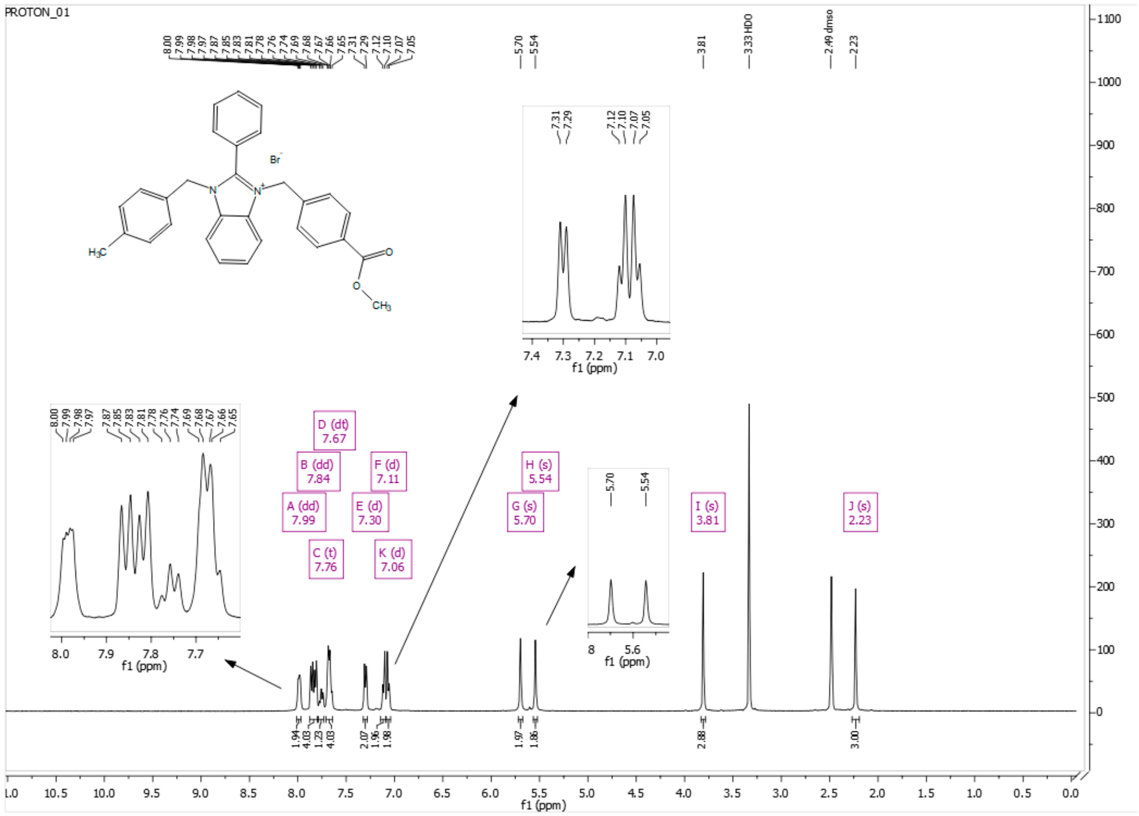

Figure S9: Compound 1e ^{1}H-NMR spectra

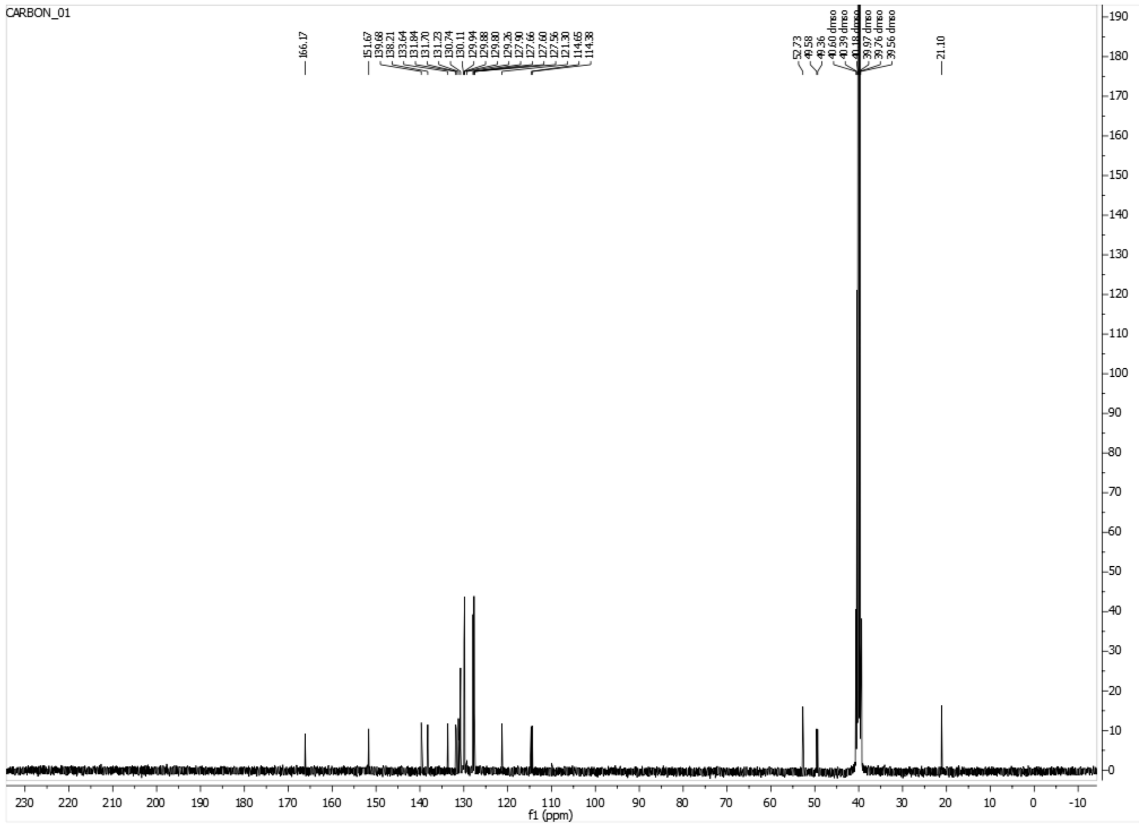

Figure S10: Compound 1e ^{13}C-NMR spectra

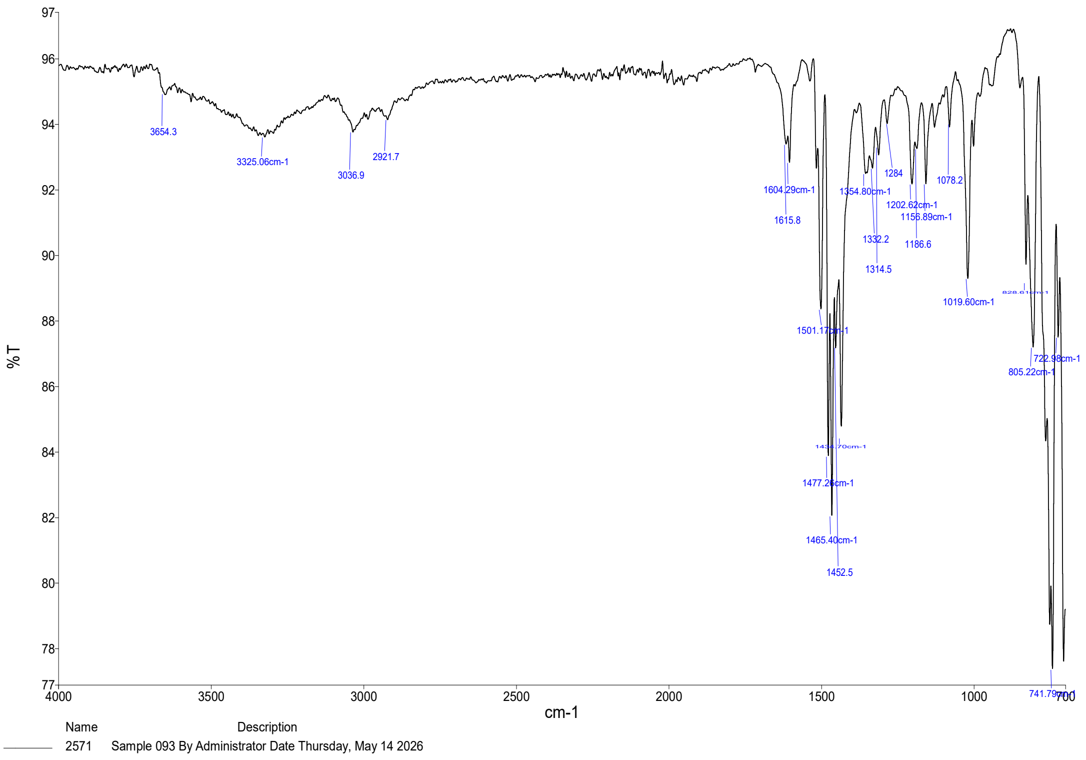

Figure S11: Compound 1a FT-IR spectra

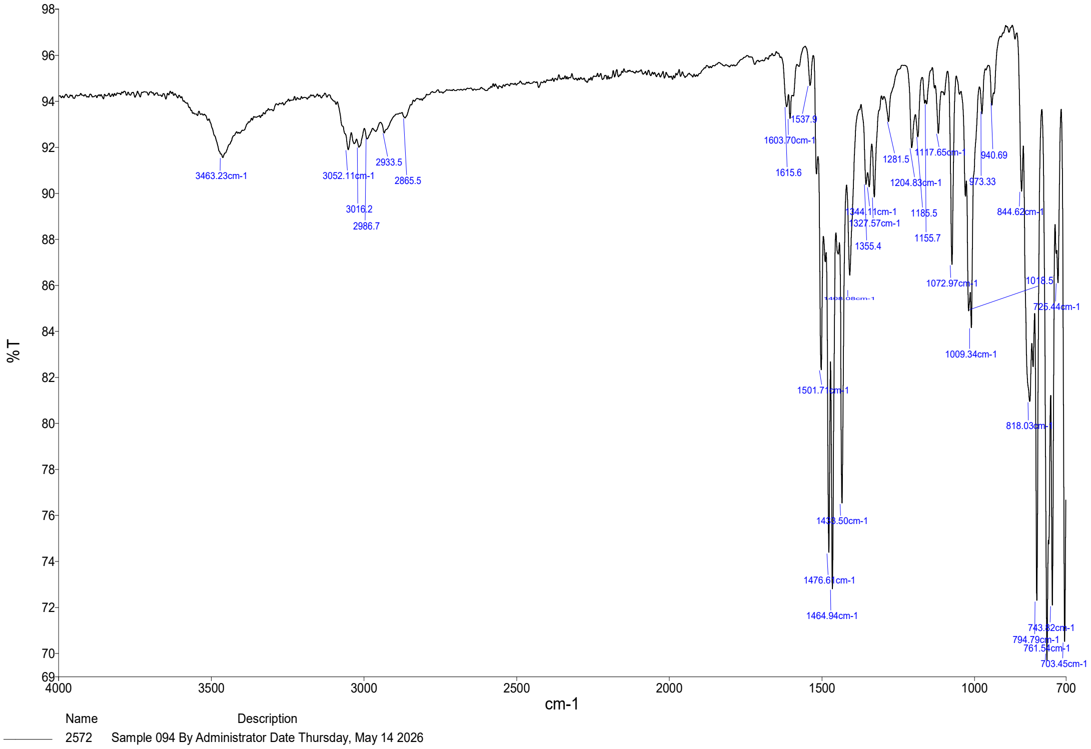

Figure S12: Compound 1b FT-IR spectra

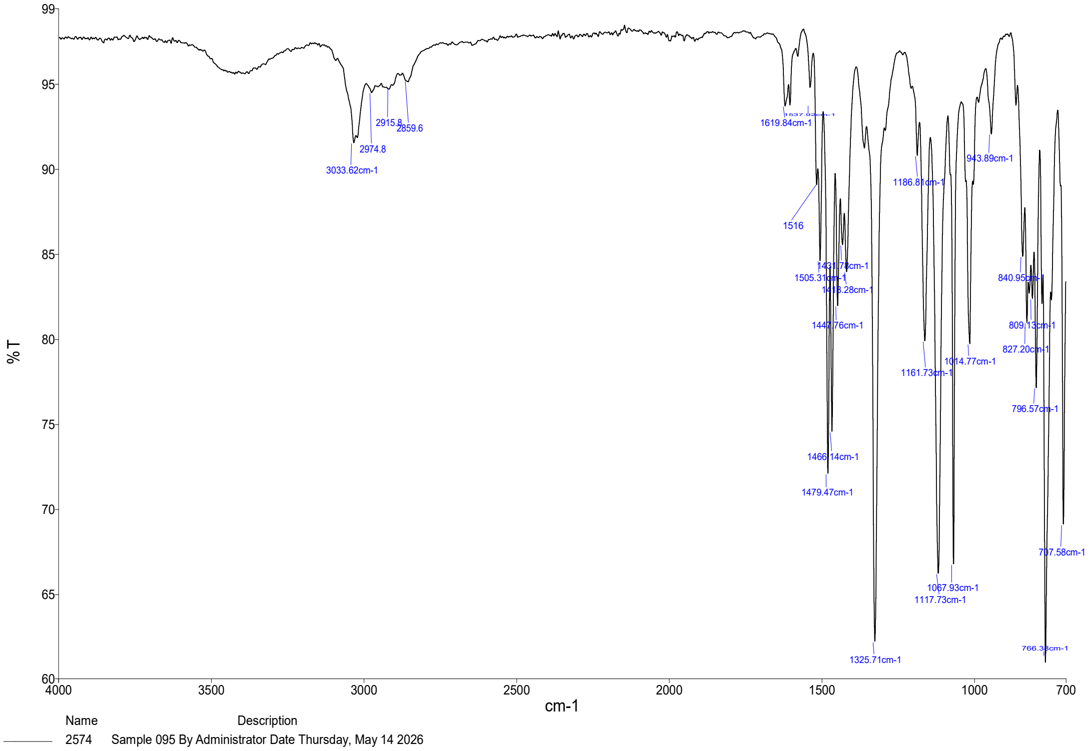

Figure S13: Compound 1c FT-IR spectra

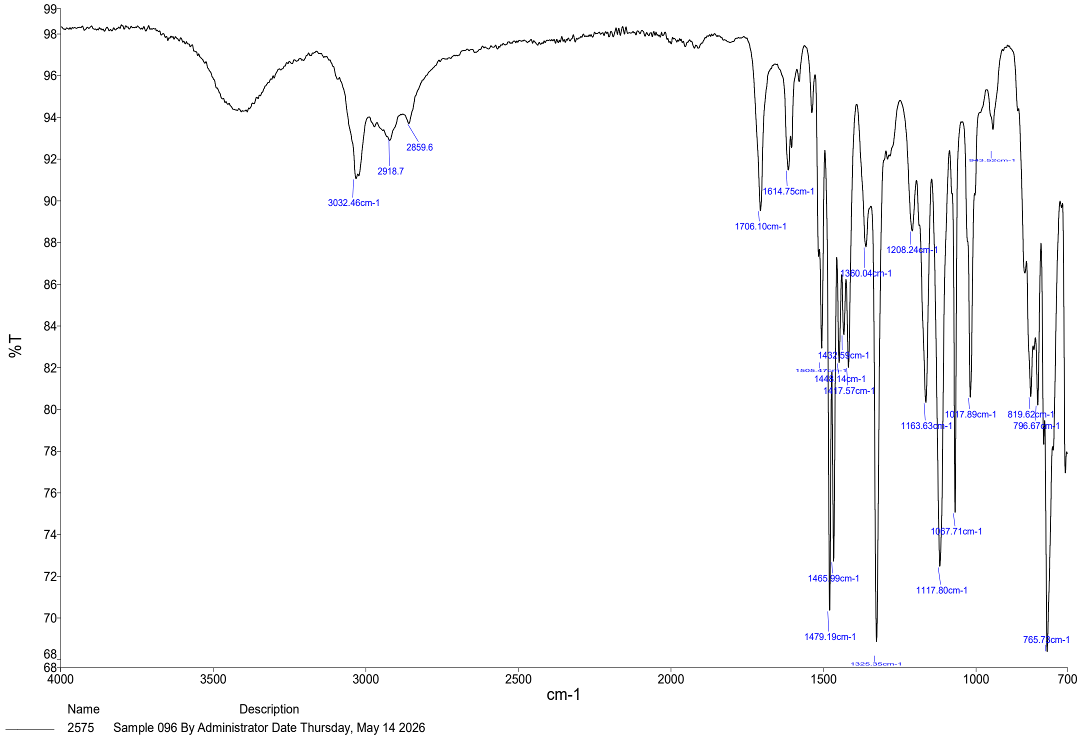

Figure S14: Compound 1d FT-IR spectra

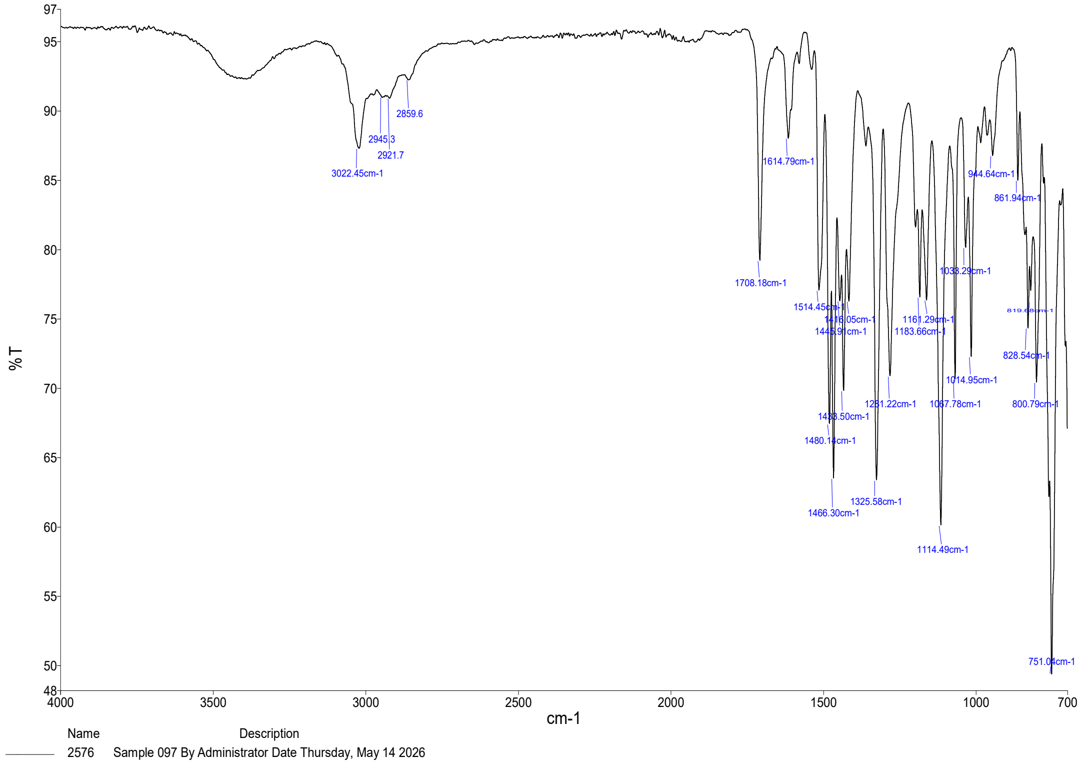

Figure S15: Compound 1e FT-IR spectra
